# Bridging the Gap: A Quality Improvement Project to Enhance Mental Health Staff Confidence in Physical Health Assessment

**DOI:** 10.1192/bjo.2026.11505

**Published:** 2026-06-30

**Authors:** Ellie Turner, Mashal Farooq, Chirag Shroff, Rajashekhar Madgula

**Affiliations:** Merseycare, Liverpool, United Kingdom

## Abstract

**Aims:**

To improve mental health staff confidence in recognising and assessing common physical health conditions (hyponatraemia, deep vein thrombosis (DVT)/pulmonary embolism (PE), cellulitis/sepsis, neuroleptic malignant syndrome (NMS), serotonin syndrome, and extrapyramidal side effects (EPSEs)) through a structured teaching programme on an acute psychiatric ward.

**Methods:**

Mental health inpatients have significant physical health comorbidities and face increased risk of acute physical deterioration. Nursing and allied health staff on psychiatric wards are often the first to notice clinical changes but informally reported low confidence in recognising and initially assessing physical health emergencies. Early identification by ward staff is crucial for timely escalation to medics and improved patient outcomes.

This quality improvement project was conducted on an acute adult mental health ward within Merseycare. A three-month teaching programme was implemented, consisting of six short (<15 minutes) educational sessions covering the above conditions. Sessions were designed for busy ward staff, adapted to areas of weakness identified on the ward, and each session was paired with a summary poster that was displayed in the clinic room to enable access across all shifts. Staff on the ward were sent a pre-teaching questionnaire and a post-teaching questionnaire to identify improvement in self-reported confidence levels.

**Results:**

10 staff members completed post-intervention questionnaire, representing a response rate of 66.7%.

Mean confidence scores across all six clinical conditions improved from 2.6 pre-intervention to 4.3 post-intervention, representing a 65.4% increase.

Individual condition improvements:

- Hyponatraemia: [2.5] to [4.2].
- DVT/PE: [3.1] to [4.3].
- Cellulitis/sepsis [2.6] to [4.4].
- NMS: [2.6] to [4.3].
- Serotonin syndrome: [2.3] to [4.4].
- EPSEs: [2.1] to [3.8].
- Confidence in escalating physical health concerns [3.9] to [4.4].
- Confidence in advocating for physical health investigations [3.7] to [4.4].

**Conclusion:**

Brief, accessible teaching sessions combined with visual reference materials significantly improved mental health staff confidence in recognising and assessing common physical health problems and emergencies within psychiatry. This low-cost, sustainable intervention addresses an important training gap in psychiatric settings and may facilitate earlier detection and escalation of physical deterioration, improving patient outcomes.

